# “Take-home” functional electrical stimulation for depression: protocol for a prototype development and proof of concept randomized controlled trial

**DOI:** 10.1186/s40814-025-01642-4

**Published:** 2025-05-03

**Authors:** Fatemeh Gholamali Nezhad, Vanessa K. Tassone, Ilya Demchenko, Jia Xi Mary Chen, Stephanie N. Iwasa, Josh Martin, Naaz Desai, Hani E. Naguib, Milos R. Popovic, Venkat Bhat

**Affiliations:** 1 Interventional Psychiatry Program, St. Michael’s Hospital, Unity Health Toronto, 193 Yonge Street 6 - 013, Toronto, ON M5B 1M4 Canada; 2 Institute of Medical Science, Temerty Faculty of Medicine, University of Toronto, Toronto, ON Canada; 3 The KITE Research Institute, Toronto Rehabilitation Institute, University Health Network, Toronto, ON Canada; 4 CRANIA, University Health Network and University of Toronto, Toronto, ON Canada; 5 Institute of Biomedical Engineering, University of Toronto, Toronto, ON Canada; 6 Krembil Research Institute, University Health Network, Toronto, ON Canada; 7 Department of Mechanical & Industrial Engineering, University of Toronto, Toronto, ON Canada; 8 Department of Materials Science & Engineering, University of Toronto, Toronto, ON Canada; 9 Li Ka Shing Knowledge Institute, St. Michael’s Hospital, Toronto, ON Canada; 10 Department of Psychiatry, Temerty Faculty of Medicine, University of Toronto, Toronto, ON Canada

**Keywords:** Major depressive disorder, Take-home treatment, Functional electrical stimulation, Facial muscles, Neuromodulation, Feasibility studies

## Abstract

**Background:**

One-third of patients with major depressive disorder (MDD) will not achieve a clinically meaningful response to available conventional treatments. More effective neurostimulation treatments are difficult to access and are associated with high hospital delivery costs. Patients would benefit from more efficacious and well-tolerated home-based neurostimulation treatments, which could be self-administered at a frequency required to treat MDD, maintain response, and reduce relapse. A potential novel intervention for MDD is bilateral functional electrical stimulation (FES) of the facial muscles. The portable FES stimulator delivers electrical current to excitable tissues and is suitable for home-based use. Based on the preliminary work demonstrating the feasibility of FES for MDD, the proposed study will develop a viable prototype for a “take-home” FES device and perform a proof-of-concept feasibility trial for participants with MDD.

**Methods:**

This is a single-site, pilot, double-blind, randomized, sham-controlled clinical trial, where 20 participants will receive 20 sessions of FES over 4 weeks. The trial will evaluate the feasibility, tolerability, and safety of home-based FES for MDD. We will also collect data on the preliminary therapeutic effects of FES on depressive symptoms and associated anxiety, quality of life, and sleep. Eligible participants will have three on-site visits including one mask development visit, one mask delivery visit, and one follow-up visit at the end of the study. They will also attend 25 online visits including a screening visit, a baseline visit, 20 days of FES treatment (sham or active), and three post-stimulation follow-up visits.

**Discussion:**

Data obtained from this trial will be used to optimize the home-based FES prototype and design a follow-up, multi-site, large-scale randomized control trial to assess the effectiveness of take-home FES. The existing evidence suggests that FES of the facial muscles can reduce MDD symptoms by enhancing positive facial feedback and altering the interoceptive bias associated with MDD, but its exact mechanism of action is still under debate. Additional trials with neuroimaging outcomes are needed to elucidate the mechanism of action of FES and the corresponding changes in the central nervous system.

**Trial registration:**

This trial has been registered at the National Library of Medicine, National Center for Biotechnology Information (ClinicalTrials.gov: NCT06261177. Registered on January 4, 2024).

**Supplementary Information:**

The online version contains supplementary material available at 10.1186/s40814-025-01642-4.

## Background

With a worldwide prevalence of 264 million people, major depressive disorder (MDD) is the most common psychiatric disorder, and heavily burdens personal and socioeconomic aspects of life [1]. Despite the significant prevalence of MDD and advances in treatment methods [2], up to 50% of patients do not achieve remission [3]. Conventional monoaminergic antidepressants are associated with significant adverse effects [4] and non-pharmacological interventions, such as psychotherapy and neurostimulation, are often difficult to access and incur high personnel and in-hospital delivery costs [5]. One way to make it easier for patients with MDD to receive treatment is to self-deliver it at home. Such patients would benefit from home-based treatments with alternative neurobiologically grounded mechanisms of action.

Bilateral functional electrical stimulation (FES) of the facial muscles is a promising novel intervention for MDD. FES has been widely researched for restoring motor function in individuals with neurological deficits, such as stroke and spinal cord injury [6–11]. Additionally, it has been shown to modulate emotions in healthy participants [3] and improve mood in patients with MDD [12]. MDD has been hypothesized to be a disorder of impaired interoception and disturbed afferent bodily signals [13]. It has been shown that certain facial expressions are associated with particular emotions (e.g., happiness, sadness, anger, and fear), which are considered universal [14]. Some facial muscle movements can be easily controlled intentionally, while others occur spontaneously during “genuine” emotional experiences. Deliberate smiles usually involve only the upward curving of the lips and are often used for social purposes without true emotional involvement. In contrast, natural smiles that result from positive emotions also involve the eyes, with the appearance of crow’s feet wrinkles and a rising of the cheeks, known as the “Duchenne marker” [15, 16]. These two types of smiles are initiated by different neural pathways: voluntary smiles come from the motor cortex and the pyramidal motor system, whereas involuntary smiles are mainly controlled by subcortical nuclei and the extrapyramidal motor system [3].

The involuntary contraction of the orbicularis oculi is often seen when people are exposed to pleasant stimuli or when they report positive subjective experiences [17]. Interestingly, it has been discovered that intentionally making and holding a facial expression can induce the corresponding emotion [15, 18]. This effect is more pronounced when a person focuses on consciously activating muscles that are typically used involuntarily, such as the “Duchenne smile” muscles [19]. Moreover, it has been demonstrated that external stimuli can also contribute to this process [20]. By consciously engaging with one’s facial expressions, it may be possible to potentiate the effect of FES, which can lead to neuroplastic changes in the brain’s emotional circuits when repeated over time [21].

Based on the principles of the facial feedback hypothesis [22], bilaterally stimulating motor units of the zygomatic major and orbicularis oculi, the “Duchenne smile” muscles of facial expression, may feed back to the critical emotional circuits of the brain involving the amygdala and emotion-to-motor transformation loop, leading to neuroplastic changes in pathways associated with positive emotion [21, 23]. FES uses an electrical current to stimulate motor units, causing the muscles to contract [24]. Zariffa et al. [3] evaluated the change in the positive and negative affect schedule-X (PANAS-X) scores in 12 healthy subjects following a single session of FES delivered to the “Duchenne” muscles. Participants reported changes in the “determined,” “daring,” “scared,” and “concentrating” scores of the PANAS-X, which suggests that emotions relevant to MDD could be modulated by FES. In a following open-label study of FES among individuals with moderate-to-severe MDD [12], participants experienced early improvements in depressive symptoms following 10 sessions of facial FES of the “Duchenne” muscles. Response and remission rates were high, and the intervention was safe and tolerable, with 50% of the sample requesting to extend their therapy and undergo an additional 30 sessions of FES (i.e., up to 40 FES sessions in total). No adverse events were reported, and compliance was high, indicating that FES is feasible, acceptable, and practical for the treatment of MDD.

As preliminary evidence concerning the feasibility and therapeutic effects of FES for MDD emerges from a single open-label study, the next logical step is to test FES among participants with MDD using a randomized sham-controlled design. Thus, this study aims to conduct a pilot double-blind randomized sham-controlled investigation of the feasibility, tolerability, and safety of 20 sessions of active FES, comprising coupled active sensory and motor stimulation of the zygomaticus major and orbicularis oculi muscles, against sham FES, comprising only sensory and non-patterned stimulation of the same muscles. Since FES devices are portable, this trial also aims to examine the potential of delivering FES as a home-based therapy; we will thus develop a prototype for a home-based device with a cast-molded personalized mask on a three-dimensional (3D)-printed mold and a programmable stimulator to enable self-administration.

## Objectives

The primary objective of this randomized controlled trial (RCT) is to provide estimates of the feasibility, tolerability, and safety of bilateral FES of the “Duchenne” muscles in participants with MDD. The outcomes include the rates of recruitment, dropout, data collection, and protocol compliance, and the number and nature of adverse events (AEs) and serious adverse events (SAEs). We aim to enhance the experience of positive emotions and treat MDD by developing a prototype for a home-based device with a personalized mask. We hypothesize that recruitment, dropout, data collection, and protocol compliance rates will not significantly differ between participants randomized to active or sham FES. Additionally, we hypothesize that the overall number of AEs and SAEs will not significantly differ between participants randomized to active or sham FES. Moreover, it is expected that the nature of AEs and SAEs that patients’ experience will not have a significant impact on the dropout and protocol compliance rates.

As a secondary objective, this study will also evaluate the efficacy of FES. Response and remission rates, as well as the sustainability of the response following the intervention, will be determined using the 17-item Hamilton Depression Rating Scale (HAM-D-17) [25] and self-rated 16-item Quick Inventory of Depressive Symptoms (QIDS-SR-16) [26] scores. Additionally, the impact of FES on anxiety, quality of life, and sleep will be assessed using the Generalized Anxiety Disorder-7 Scale (GAD-7) [27], World Health Organization-5 Well-Being Index (WHO-5) [28], and Pittsburgh Sleep Quality Index (PSQI) [29].

## Methods

### Study design and setting

**Fig. 1:**
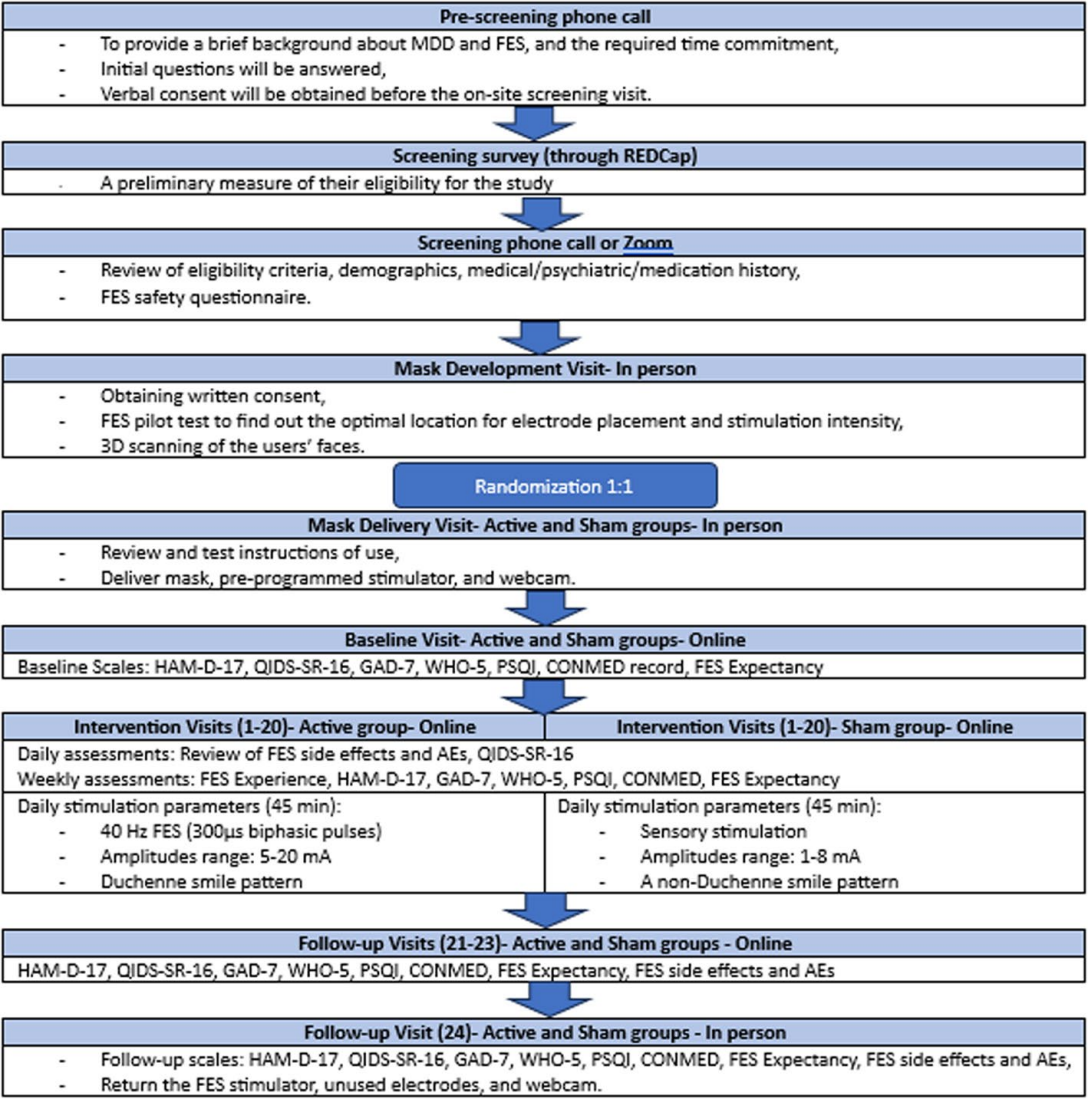
Study flow diagram

**Fig. 2:**
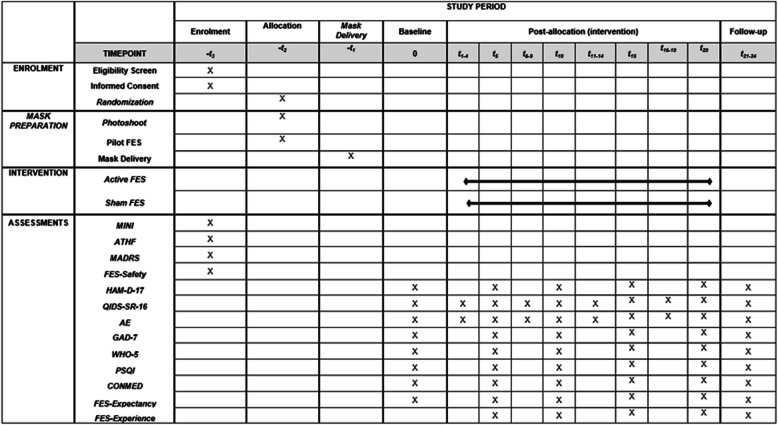
SPIRIT figure

This study is a single-site, pilot, double-blind, randomized, sham-controlled clinical trial. Eligible participants enrolled in this clinical trial will have a total of 28 study visits. There will be 3 on-site visits (including one mask development visit, one mask delivery visit, and one follow-up visit at the end of the study) taking place at St. Michael’s Hospital, Unity Health Toronto. Twenty-five online visits (including the screening visit, baseline visit, 20 sessions of active or sham FES, and 3 post-treatment follow-up visits) will take place over the phone or video conference on the Zoom platform. Figure 1 describes the study flow. Figure 2 provides the SPIRIT figure as this study protocol adheres to the SPIRIT framework [30]. The SPIRIT Checklist is available in additional file 1, and the full protocol (version 3, dated on January 22, 2024) is available in Additional file 2.

### Participants

The first means of recruitment is through a referral process. Recruitment will occur at the Interventional Psychiatry Program at St. Michael’s Hospital. Twenty participants with the diagnosis of MDD with ≤ 2 failed treatment trials (i.e., non-treatment-resistant depression), as determined by a physician and validated by a mini-international neuropsychiatric interview (MINI) [31], will be recruited. This will include English-speaking males and females aged > 18 years with a diagnosis of unipolar, non-psychotic current major depressive episode. Eligible participants will have a Montgomery–Åsberg Depression Rating Scale (MADRS) [32] total score of ≥ 7 with no active suicidal ideation (as demonstrated by a score of ≥ 2 on MADRS item 10). As part of the inclusion criteria, participants will be asked about their medical comorbidities and concomitant medications. To confirm eligibility, they are required to have no change in their medication regimen or other forms of treatments (e.g., psychotherapy) for at least 4 weeks (28 days) prior to beginning the study, during the 20-session treatment period, and during the 4-week post-treatment observation period. This will be established through self-report, in combination with the Anti-depressant Treatment History Form (ATHF) [33] filled out by the participant. All the participants will be recruited from the Interventional Psychiatry Program at St. Michael’s Hospital, Unity Health Toronto, Canada. The full consent form (dated on January 22, 2024) is available in Additional file 3.

Participants will be excluded if they have active substance abuse or dependence (with the exception of nicotine and caffeine) or a history of epilepsy or seizures. Having cancer, radiation, or botulinum toxin injection into muscles targeted by the stimulation in the past 6 months is also part of the exclusion criteria. Other exclusion criteria include any paralysis of facial nerves, metallic implants in the mouth or metal braces near the potential sites of electrical stimulation, any type of implanted electronic devices, and current fibromyalgia. Individuals who are currently receiving or have received repetitive transcranial magnetic stimulation within the last month (28 days) before screening and pregnant females will also be excluded.

### Screening and consent

Individuals who are referred by a mental health care provider will be contacted for an initial pre-screening over the telephone. During the telephone pre-screening, researchers will provide a brief background about MDD and FES. The researcher will then ask for verbal consent and the participant’s email address. Interested participants will access a screening survey through Research Electronic Data Capture (REDCap) via a link which will be sent to their email. The survey will be a preliminary measure of their eligibility for the study. Once a participant has completed the REDCap screening form, participants who appear eligible will receive another telephone call to schedule the screening visit. The screening visit will take place on the phone or via a Zoom meeting. During the screening visit, the eligibility criteria and medical, psychiatric, and medication history will be reviewed.

If the subject satisfies the inclusion criteria, the mask development and delivery visits will be scheduled to be carried out on-site. Written informed consent will be obtained during the first on-site visit (mask development visit). The mask development and delivery process are described in the following sections.

### Sample size

For this pilot feasibility trial, we aim to recruit 20 participants in total. The size of the sample is consistent with neurostimulation feasibility trials in MDD [12, 34–36] and the detection of a moderate or large treatment effect size (*f* ≥ 0.25) with 80% power. Study participants will be recruited from the Interventional Psychiatry Program (IPP) at St. Michael’s Hospital, which annually receives approximately 100 referrals. Based on the past data, we expect that 40% of referrals are eligible for inclusion in the trial and we could have a participation rate of 60% from those eligible patients. Therefore, we could recruit 24 participants in a year. Thus, to meet our target sample size (10 for the active arm and 10 for the sham) and consider any unforeseen recruitment delays, a 12-month recruitment period will be sufficient. As we are primarily interested in precise estimates of tolerability, outcome variability, and preliminary effectiveness of active FES compared to sham FES in patients with MDD, utilizing a sample size of 20 allows us to estimate the participation rate of 60% with a 95% confidence interval ranging from 39% to 78% (https://www.statskingdom.com/proportion-confidence-interval-calculator.html). Therefore, this approach not only assesses the operational aspects of conducting this trial, but the study results will aid in the planning of a larger, sufficiently powered efficacy trial.

### Randomization and masking

The target recruitment time is 12 months. Participants will be randomized in a 1:1 allocation to receive FES (active pattern) or FES (sham pattern). Block randomization will be generated using a secure online randomization module on REDCap, and the samples will be stratified for sex, given the potential differences in response and tolerability in these groups. A research member will generate and administer the randomization schedule and will be instructed not to divulge group assignments to the participants to allow for concealment.

Participants and most study staff will be blinded. Only the physical therapist and the trained study staff member, who generated the randomization schedule, will be unblinded to monitor the participant. Measures taken to prevent functional unblinding include sensory and non-patterned sham stimulation and delivering automated messages saying “smile” to both active and sham groups. We will also evaluate each participant’s ability to discriminate which treatment they received after each session to assess blinding effectiveness.

Emergency unblinding will only occur when knowledge of the intervention is essential for participant care as determined by the principal investigator. In the event of an emergency unblinding of one or more research team members, the timing, reason, and personnel involved will be recorded and blinding will be maintained in as many other study personnel as possible.

### Mask development and delivery process

**Fig. 3:**
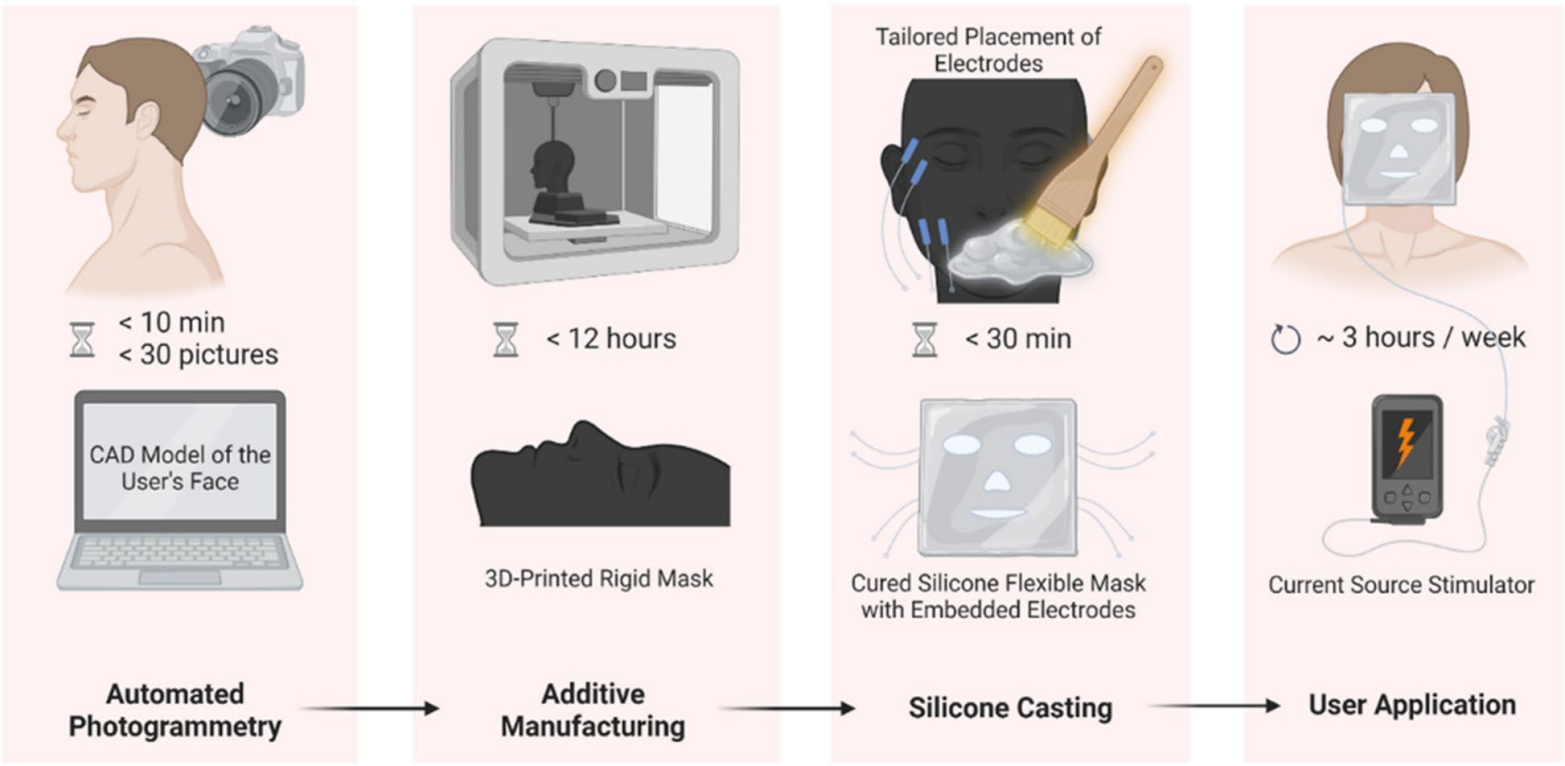
Mask preparation process (created with BioRender.com)

The manufacturing of the mask will need to be personalized for each participant so that the orbicularis oculi and zygomaticus major muscles can be properly targeted for electrical stimulation. To customize the masks, an effective and convenient 3D scanning technique based on photogrammetry will be used as a preliminary step to obtain the surface data for the facial features of each person for this purpose. For each person, an average of 50 pictures will be taken, covering mainly the face and head areas from various perspectives through sweeps of horizontal and vertical angles. Results from the scanning, in 3D mesh formats, will be processed by means of computer-aided design (CAD) software packages to modify the geometry, remove undesired areas, add required features for integrating electrodes, cabling, and fasteners, and finally export the CAD file to a 3D printable format in STL file. The mask preparation process is presented in Fig. 3.

**Fig. 4:**
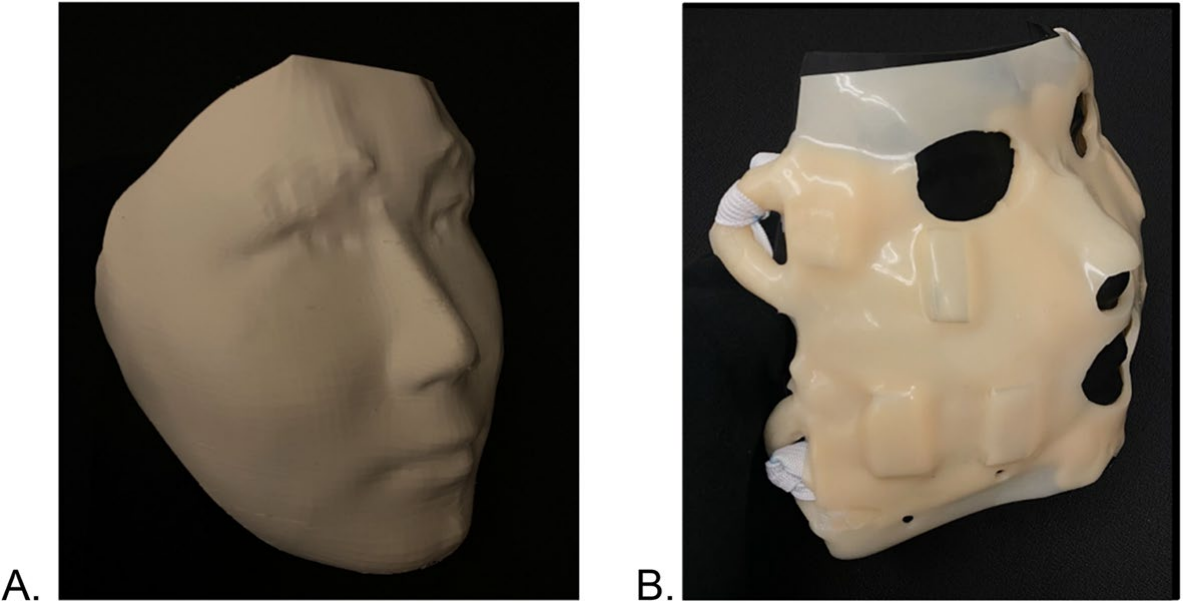
**A** Customized mask mold: 3D-printed with polylactic acid filaments. **B** Customized flexible mask: flexible platinum-cured silicone with indents for FES electrodes

A rigid plastic model of the patient’s face will subsequently be 3D printed (Fig. 4A) to be used as the mold to cast the personalized flexible mask made of food-grade, skin-safe platinum-cured silicone rubber. After printing the rigid mold, we will cast the flexible mask (Fig. 4B) with embedded slots for attaching fasteners to be used to attach the electrodes to the desired locations of the mask surface facing the skin and then run the cables for connection to the stimulation system. The placement of the electrodes will be fine-tuned during the first on-site visit while the mask preparations and pilot FES are being done.

For the purpose of finding the optimal location for electrode placement, single-use commercially available FES electrodes (MyndTec) made of skin-safe self-adhesive hydrogel and conductive carbon will be placed on the face, and a pilot FES trial will be carried out to stimulate their orbicularis oculi muscle and zygomaticus major muscles. To gather the proper electrode placement locations to induce the desired facial expression, 8 electrodes will be placed on the face: 2 electrodes under the left eye, 2 electrodes under the right eye, 2 electrodes on the left cheek, and 2 electrodes on the right cheek. The exact locations of these electrodes might differ based on the induced muscular response. The final placement will induce proper contraction with minimal FES current and minimal reported discomfort. The entire process takes approximately 20–30 min. Once the electrode locations are optimized, a picture will be taken so that the electrode placement will be adjusted accordingly on the personalized mask.

### Intervention

The intervention visits (visits 1–20) will occur online over the Zoom platform. All the stimulation parameters are pre-programmed to the FES device, and intensity level is the only value that needs to be adjusted for comfort and function by the patient. Amplitudes will be individually determined at the beginning of each session to achieve visible contractions in the target muscles while avoiding unnecessary pain or excessive movement, such as complete eye closure. The values for sensation and contraction vary between individuals and thus need to be personalized. Previous studies on FES of facial muscles [3, 12] have used a range of 1–15 mA; however, these studies did not include a sham arm. Given that we are using a different stimulator, with varying electrode sizes and considering individual differences in sensitivity to stimulation, we conducted multiple test sessions with approximately 10 volunteers of diverse facial configurations, genders, and skin sensitivities. These tests determined that all volunteers experienced the required sensation for the sham group at or below 8 mA, and all achieved the desired muscle contraction for the active arm without pain at intensities between 1 and 25 mA. Therefore, we selected 1–8 mA for the sham FES group and 1–25 mA for the active FES group as intensities, as these ranges meet the objectives of stimulation for each study arm. Additionally, a physical therapist or a trained study staff member will monitor the participant during the intervention visits.

#### Active group

**Fig. 5:**
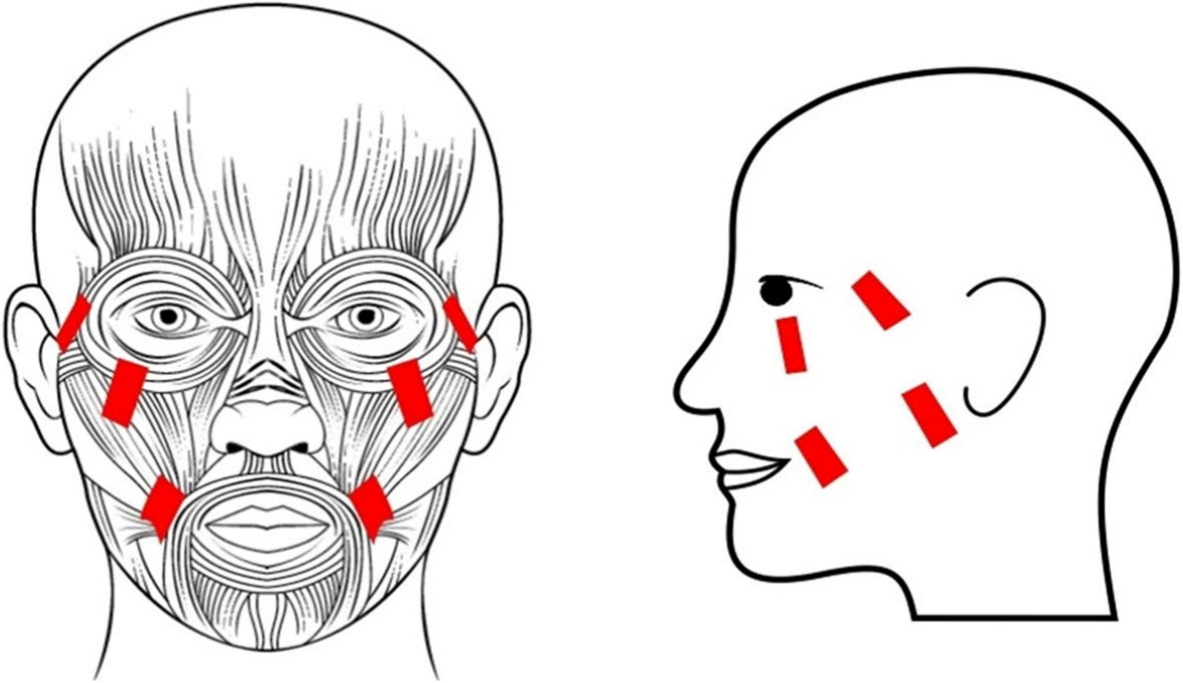
Surface electrode placement for the functional electrical stimulation of bilateral zygomaticus major and orbicularis oculi muscles for the treatment of major depressive disorder

Participants allocated to the active arm will receive simultaneous transcutaneous FES therapy of the bilateral zygomaticus major and orbicularis oculi muscles, which produces an expression of happiness according to the facial action coding system [37]. Surface self-adhesive electrodes in the mask will be placed bilaterally on the zygomaticus major and orbicularis oculi muscles (Fig. 5). FES will be delivered simultaneously to all four muscle groups using the Twin Stim® Plus Digital TENS/EMS stimulator and the developed mask. Each FES intervention visit will last 60 min and includes 15 min of preparation and 45 min of stimulation. FES stimulation parameters to be used are 300 μs long charge-balanced biphasic pulses delivered at 40 Hz, with amplitudes in the range of 1–25 mA to activate muscles, with alternating 15-s periods of stimulation and rest [38].

Synchronized stimulation of four muscles (i.e., bilateral zygomaticus major and bilateral orbicularis oculi) will be delivered in a 15-s ON and 15-s OFF cycle. Automated messages to voluntarily “smile” will be presented and precisely timed with the FES-related facial stimulation (i.e., only during 15-s ON) and delivered over speakers.

#### Sham group

Participants recruited to the sham arm will also receive FES of the facial muscles following the same setup as the experimental group. However, these participants will only receive sensory stimulation (1–8 mA stimulation, to induce the feeling of the stimulation, with other parameters the same as in the active arm) in a non-Duchenne smile pattern.

### Outcome measures and data collection

The primary outcomes for this study will be the feasibility, tolerability, and safety outcomes that include recruitment, dropout, data completion, and protocol compliance rates, as well as the number and nature of AEs and SAEs. Secondary outcomes will also include the (A) changes in depression symptoms, measured by the HAM-D-17 scale and the QIDS-SR-16; (B) changes in anxiety, measured by the GAD-7; (C) changes in quality of life, measured by the WHO-5; (D) changes in subjective sleep, measured by the PSQI; and (E) HAM-D-17 response and remission rates and sustainability of effects for up to 4 weeks (28 days) following the intervention. All the self-report questionnaires will be completed through REDCap, and an independently trained rater will administer the psychiatric interview.

An independently trained rater will assess depressive symptoms using the HAM-D-17 at baseline (visit 0), after every five treatment days (visit 5, visit 10, visit 15, and visit 20), and during the post-stimulation visits (visits 21 to 24). Additionally, subjects will be queried each day about their experience and the AEs and SAEs they might experience. A participant “FES Experience” assessment will be completed at the end of each week (visits 5, 10, 15, and 20) via video conference to monitor participant burden.

The independent rater will be blinded to the treatment being administered. Participation in the trial will be discontinued if the participant develops active suicidal intent, attempts suicide, or experiences worsening of depression as defined by an increase in the QIDS-SR-16 score from baseline by more than 25% during two consecutive assessments.

### Medical record data collection

For this research study, required personal health information includes name, date of birth, new or existing medical records that include psychiatric or medical conditions, current and past medications, presence of surgical implants, and illnesses or psychiatric procedures that may influence the ability to participate in the study. Some personal health information such as consultation reports and medical history may be kept as source documents. This information will be obtained from the subject, his/her physician, or his/her medical health record. The case report forms (CRFs) will not contain any personal health information. Only the subject number will be recorded in the CRF.

### Adherence to intervention and data collection requirements

FES stimulation during pregnancy has no known risks to the ova or fetuses; however, there is always a possibility that if participants are pregnant, FES stimulation may have risks that we do not know about. To prevent having missing data due to pregnancy and to consider safety aspects, appropriate family planning methods will be discussed with female participants. Additionally, non-English-speaking individuals will be excluded from this RCT because the ability to accurately and completely communicate study information, answer questions about the study, and obtain consent is necessary.

### Adverse event monitoring

AE data will be collected from the start of the experimental protocol to the end of study participation. All AEs, regardless of attribution to the FES or pre/post assessments, will be collected and recorded using a standard AE form. Participants will be asked, in an open-ended way, about the presence of any such AEs on a daily basis. Additionally, a standard questionnaire for FES-related AEs will be completed in the period after every FES session. The intensity of each AE will be graded as mild, moderate, or severe. If an event occurs that is not expected, the study doctor of medicine (or covering investigator) will be informed in real time to assess the event, advise on immediate care of the participant, and determine the necessary reporting steps. Any events that are serious or unexpected in nature, severity, or frequency as compared to the risks described in the study plan will be reviewed by the principal investigator or designee to determine the relationship of the event to the study. Reportable events will be submitted to the Unity Health Toronto Research Ethics Board per determined policies.

### Patient retention methods

For each study visit, the research staff will give each participant a reminder call/email and, during the study, research staff members will be easily available for the participants to contact via email or phone. We will aim to have a specific research team member assigned to complete all sessions with the same participant. Participants will be reimbursed for the in-person visits.

### Data management and quality assurance plan

Quality assurance methods will be rigorously implemented for a study launch, starting with a start-up meeting involving the principal investigator and all site personnel. Additional training sessions will precede the study, focusing on thoroughly explaining procedures and data completion. Active data will be securely stored on the REDCap server, accessible only to the project administrator for analysis. Compliance with data protection laws will be ensured. Research staff will have access to source documents, and data quality will be maintained through filters and accuracy checks by a second reviewer. All trial records will be retained for 7 years according to Unity Health requirements.

### Data analysis plan

Initial analyses will summarize socio-demographic and clinical characteristics by treatment group. Primary analyses will be performed on the modified intention-to-treat (ITT) population, including all randomized participants who received at least one FES treatment. The feasibility outcome defined as overall dropout rates will be summarized using descriptive statistics such as counts and percentages. Feasibility analyses will report feasibility outcomes using descriptive statistics with counts and proportions for categorical data and mean and standard deviations or median and interquartile range, as appropriate, for continuous data. The comparison of the proportion lost to follow-up in the active and sham groups will be estimated along with a 95% confidence interval. The proportion compliant with the protocol, including treatment compliance as well as study completion and complete data on clinical outcomes will also be estimated with a 95% confidence interval. The blinding index proposed is scaled to an interval of − 1 to 1, 1 being a complete lack of blinding, 0 being consistent with perfect blinding, and − 1 indicating opposite guessing, which may be related to unblinding. The success of blinding patients will be assessed by computation of Bang’s blinding index.

The preliminary effectiveness analysis will report the between-group difference in change from baseline to treatment visit 20 in the HAM-D-17 and its 95% confidence interval, using analysis of covariance, with treatment visit 20 HAM-D-17 as the outcome and baseline HAM-D-17 and stratification variables (sex) as covariates. We will also report estimates for the standard deviation of the HAM-D-17 score and the within-person correlation between baseline and follow-up HAM-D-17 scores. A similar approach will be used for all other continuous variables for secondary outcome measures (e.g., QIDS-SR, GAD-7). Differences between groups in dichotomous outcomes (e.g., response, remission, adverse effects) will be described using an odds ratio and 95% confidence interval. To explore the sustainability of the treatment effect, we aim to also report changes in HAM-D-17 scores during the month after 20 treatments. Sex- and gender-stratified analysis will be carried out.

### Participant withdrawal

Participants are free to withdraw their consent and stop research study participation at any time without penalty or loss of benefits to which they are otherwise entitled. A participant’s doctor may withdraw them from the study if they feel that it is in their best interest. The principal investigator may also decide to terminate the study at any time if the stimulation device is believed to be unsafe.

A study participant will be discontinued from further participation if they fail to adhere to the study procedure and/or miss more than two treatment visits and/or meet any exclusion criteria. Additionally, if a participant withdraws or is withdrawn from the study, they will be asked questions about their experience with the FES stimulation. They will also be asked to cooperate with whatever laboratory tests or medical examinations the doctor considers necessary. We will collect safety data on any participant discontinued because of an AE or SAE. If an AE has been reported, researchers will help the participant seek the medical care they need, and a follow-up will be performed by the principal investigator. In the case of an early withdrawal, the researcher will make a note to file indicating this.

### Success criteria

The success of the feasibility study will be assessed quantitatively in relation to the overall recruitment rate, dropout rate, data completion, and the number and nature of AEs and SAEs. The recruitment rate will be calculated based on the final number of randomized individuals divided by all individuals assessed for eligibility. A recruitment rate of 45% to 60% or more will be considered a success in recruitment. The dropout rate will be calculated from the number of randomized participants who do not complete the study, with a target of 20% dropout or less to indicate successful adherence. Regarding data completion, the completeness of outcome measures will be tracked. We expect that 85% or more of the participants will complete all outcome measures by follow-up visit 4. Additionally, after each treatment visit, adverse events will be noted for both treatment groups using the FES adverse events screening form. Each reported AE will be classified into mild, moderate, or severe, and the principal investigator will determine whether they are serious or non-serious. Additionally, adverse events will be noted at each follow-up visit to evaluate the sustainability of each AE by tracking the resolution or progression of AEs. The number of AEs in each category as well as their duration will be reported. We anticipate that the number and nature of AEs and SAEs will not be different between active FES and sham and will resolve by the fourth follow-up visit.

If the feasibility of this study is approved, a larger scale RCT can be conducted. If any of these feasibility objectives are not satisfied, we will suggest modifications to the protocol and study design. We will explore what additional modifications could be made and whether the trial is feasible if one or more of the criteria are not met.

## Discussion

Data obtained from this trial will be used to optimize the home-based prototype and design a follow-up, multi-site, large-scale RCT to assess the effectiveness of take-home FES. The existing evidence suggests that FES of the facial muscles can reduce MDD symptoms by enhancing positive facial feedback and altering the interoceptive bias associated with MDD, but its exact mechanism of action is still under debate. Additional trials with neuroimaging outcomes are needed to elucidate the mechanism of action of FES and the corresponding changes in the central nervous system.

While MDD is the leading cause of disability, and currently available treatments are associated with side effects and low adherence [39], FES, as a well-established treatment method in people with neurological deficits [40], can be a promising treatment method for MDD [12]. Pilot feasibility studies with FES among healthy participants [3], followed by repetitive FES for MDD participants in an open-label mixed-methods study [12], informed this study. The promising outcomes achieved from the open-label study for MDD [12] showed that FES was well-tolerated, with the potential to be administered with limited physician oversight. Thus, we set out to develop a prototype for a personalized mask for FES for depression and to perform a proof-of-concept clinical trial that assumes FES might be a potentially efficacious and well-tolerated home-based neurostimulation treatment that can offer an alternative therapeutic modality for MDD. This RCT is primarily interested in precise estimates of feasibility, tolerability, safety outcome variability, and preliminary effectiveness of active FES compared to sham FES in patients with MDD that will aid in the planning of a larger, sufficiently powered efficacy RCT.

The comparison of the overall dropout and protocol compliance rates and the number and nature of AEs and SAEs between active and sham groups will demonstrate how feasible, tolerable, and safe self-administered FES is for this population. Additionally, comparing the changes in total scores of the HAM-D-17 and QIDS-SR-16 in the active and sham groups over 4 weeks will demonstrate the primary efficacy outcomes for this prototype in MDD. Moreover, the total scores obtained from pre- and post-stimulation administration of the GAD-7, WHO-5, and PSQI over four weeks will reveal differences in the response and remission rates in active and sham groups following self-administered FES.

### Potential benefits

Participants in this study may experience some degree of relief from mood symptoms as a result of the FES intervention. The chance to understand and develop a new treatment for a wide range of psychiatric disorders is an important step in helping the millions of people in the world who suffer from the same condition.

### Potential risks

There are no serious risks to the participants associated with the treatment used in this study. No safety concerns or SAEs have been reported for FES except for common mild AEs including temporary redness below the area of the electrodes and muscle fatigue or soreness [41]. An AE self-report and adverse event log will be maintained as part of the study and systematically completed by the study coordinator. Moreover, the materials used for printing the FES mask are chosen to be biocompatible and chemically stable. Therefore, there is not expected to be any skin sensitivity to the mask’s material. However, we will monitor the user’s face after experiencing multiple courses of stimulation to ensure that there is no skin sensitivity or discomfort. The CAD design can be adjusted to address any observed discomfort or geometrical mismatch when participants put on the mask.

### Further directions

Different mechanisms of action have been used to explain the effects of FES in populations with neurological deficits, such as stroke and spinal cord injury. Using functional and structural brain imaging methods would be necessary to assess how the brain’s form and function change following FES. Future studies can benefit from using neuroimaging alongside clinical assessments to better understand the FES mechanisms of action.

### Dissemination policy

We aim to publish our results in high-impact factor peer-reviewed journals and present them at relevant national and international conferences. 

## Supplementary Information

Additional file 1.

Additional file 2.

Additional file 3.

## Data Availability

For this protocol, no data has been generated or analyzed. To enable trial-related monitoring, audits, and inspections—in accordance with participant consent—host institution and regulatory authorities will be given direct access to paperwork and materials. In accordance with the instructions provided by the study’s funding source (Connaught Innovation Award), the data management plan that is created prior to the study’s start will specify how and where the study protocol and data gathered will be made available.

## Abbreviations

AE: Adverse events
ATHF: Antidepressant treatment history form
CAD: Computer-aided design
CRF: Case-report form
FES: Functional electrical stimulation
GAD- 7: Generalized Anxiety Disorder-7 Scale
HAM-D- 17: 17-Item Hamilton Depression Rating Scale
ITT: Intention-to-treat
MADRS: Montgomery–Åsberg Depression Rating Scale
MDD: Major depressive disorder
MINI: Mini-International Neuropsychiatric Interview
PANAS-X: Positive and Negative Affect Schedule-X
PSQI: Pittsburgh Sleep Quality Index
QIDS-SR- 16: Self-rated 16-item Quick Inventory of Depressive Symptoms
REDCap: Research Electronic Data Capture
RCT: Randomized controlled trial
SAE: Serious adverse events
WHO- 5: World Health Organization-5 Well-Being Index
3D: Three-dimensional
