# Supplementary material for: “Take-home” functional electrical stimulation for depression: protocol for a prototype development and proof of concept randomized controlled trial
Source: Pilot Feasibility Stud. 2025 May 3;11:60. doi: 10.1186/s40814-025-01642-4 (PMC12049065; doi:10.1186/s40814-025-01642-4)
Supplement: Supplementary file 3 — Additional file 3. [file 40814_2025_1642_MOESM3_ESM.pdf]

UNITY HEALTH TORONTO

Letter of Information and Consent to Participate in a Research Study

**Study Title:**	**"Take-Home" Functional Electrical Stimulation for Depression: Prototype Development and Proof of Concept Clinical Trial**
**Principal Investigator:**	**Dr. Venkat Bhat, MD MSc FRCPC DABPN**<br>Director, Interventional Psychiatry Program, St. Michael's Hospital, Unity Health Toronto<br>Assistant Professor, Department of Psychiatry, University of Toronto<br>Phone: (416) 864-6060 x76404, Fax: (416) 864-5996
**Co-Principal Investigator:**	**Milos R Popovic, PhD**<br>Director, KITE Research Institute, Toronto Rehabilitation Institute, University Health Network<br>Professor, Institute of Biomedical Engineering, University of Toronto Phone: (416) 597-3422 x7628<br><br>**Hani Naguib, PhD**<br>Professor and Director, University of Toronto<br>Phone: (416) 978-7054<br><br>**Wendy Lou, PhD**<br>Professor, Division Head of Biostatistics, Dalla Lana School of Public Health, University of Toronto
**Study Coordinator:**	**Fatemeh Gholamali Nezhad**<br>Postdoctoral Fellow, Interventional Psychiatry Program, St. Michael's Hospital, Unity Health Toronto<br><br>**Ilya Demchenko**<br>Research Coordinator I, Interventional Psychiatry Program, St. Michael's Hospital, Unity Health Toronto
**Study Funder:**	Connaught Innovation Award, University of Toronto

Conflict of Interest Statement:

St. Michael's Hospital is receiving financial payment from the funders to cover the cost of conducting the study. The Principal Investigator of this study has no financial or competing interests to declare.

Dr. Popovic is involved with MyndTec Inc. (www.myndtec.com) as a shareholder and a director of the company. As a director, Dr. Popovic receives financial compensation for his time on the activity. MyndTec is presently manufacturing the product MyndMove. This is a Functional Electrical Stimulation device for restoring upper limb function in people with upper limb paralysis. Functional electrical stimulation is what we are doing in this study.
Dr. Popovic has a patent approved on February 16, 2016, with Dr. S. Hitzig and Dr. J Zariffa "Functional electrical stimulation method, use and apparatus for mood alteration". The applicant is the University Health Network. Mood alteration is what we are doing in this study.
Dr. Popovic is consulting for a company called Fourier Intelligence which is designing rehabilitation robotics technologies and receives financial compensation for this activity.

STUDENT PROJECT DECLARATION:

This study is a student project conducted by Dr. Fatemeh Gholamali Nezhad, a postdoctoral fellow at St. Michael's Hospital, Unity Health Toronto, as part of her fellowship requirements at the academic institution. This study is supervised by Dr. Venkat Bhat, director of the Interventional Psychiatry Program St. Michael's Hospital, Unity Health Toronto.

Introduction:

You are being asked to consider participating in this research study because you have a diagnosis of Major Depressive Disorder (MDD) and are interested in considering take-home Functional Electrical Stimulation (FES).

All research is voluntary – you do not have to participate, and you can withdraw at any time.

Before agreeing to take part in this research study, it is important that you read the information in this research consent form. It includes details we think you need to know in order to decide if you wish to take part in the study. If you have any questions, ask an investigator or a research team member.

You should be aware that it is possible that the St. Michael's Hospital study investigator will also be your treating doctor.

If you choose to participate in the study, you will need to sign this Letter of Information and Consent form. You should not sign this form until you are sure you understand the information. You may also wish to discuss the study with others, such as your friends, family, and/or family doctor.

Background and Purpose of the Research Study

The purpose of this study is to learn whether Functional Electrical Stimulation (FES) of the facial muscles is effective in treating Major Depressive Disorder (MDD) and to develop a model (personalized mask and programmable stimulator) for take-home delivery.

A potential new intervention for treating MDD involves stimulating specific facial muscles with an FES device. Applying FES on specific facial muscles related to smile patterns may result in better control of emotions and mood elevation. FES is a low-risk, non-invasive technique which causes muscles to contract using electrical current. Here we will be applying FES to specific facial muscles. This electrical current may cause gradual, long-lasting changes in brain activity levels due to the physiological connection existing between facial muscles and the brain. This may be useful for treating disorders affecting one's thoughts, emotions, and behaviour. There is also a need for "take-home" electrical stimulation methods, which has been heightened due to COVID-19. The project will aim to address an important problem - the need for home-based treatment for MDD in pandemic/non-pandemic contexts.

FES is widely used in clinics for motor function improvements in individuals with neurological impairments such as stroke and spinal cord injury. A study that applied FES to the facial muscles of 12 healthy individuals provided evidence that FES may be able to modulate mood when compared to 12 healthy individuals that did not receive FES. Another study on 10 participants with MDD showed general improvements in participants' symptoms of depression and insomnia after 10 FES sessions. Moreover, this small-scale preliminary study with 10 participants with MDD has shown that FES may be a safe and feasible treatment option for MDD with minimal risks and side effects. The approach used in this study, FES, has been well studied over the past several decades, but its effects have not yet been confirmed in treating psychiatric disorders.

This study will examine the feasibility of a take-home FES intervention and will investigate the effectiveness of FES as a potential treatment for MDD. The findings resulting from this study will help to guide future studies in assessing the effectiveness of FES in a larger number of participants.

The Twin Stim® Plus Digital TENS/EMS stimulator that will be used in this study is a Canadian licensed medical device that is used for pain relief in patients who suffer from chronic pain. Its use in this study for MDD is off-label and is considered exploratory.

Research Study Design and Duration

Study Design

This is a pilot study that will aim to develop a viable model for a "take-home" FES device and looks at the feasibility, safety, and tolerability of FES. The results of this study will help us plan a future study looking at whether FES can be an efficacious treatment strategy for MDD. We will perform a trial for 20 participants with MDD over 12 months.

If you decide to participate in the study, you will be asked to come into the hospital three times: once for the mask development session where a personalized portable mask will be designed based on your individual facial features, once where you will pick up your mask, electrodes and the stimulator, and once to do the final follow-up and drop off the stimulator. You will then self-deliver FES stimulation (45 minutes/day+ 15 minutes for preparations) at home over 20 days with remote monitoring/support.

The study is a double-blind, randomized, sham-controlled trial. This means that:

- In this study, there will be two groups of participants:
  - One group will receive “active” FES stimulation.
  - The other group will receive “sham” stimulation. If you are assigned to this group, you will not receive any “active” FES stimulation.
- Neither you nor most of the study staff will know which group you are assigned to. In the event of a medical emergency, the study doctor will be allowed to find out which group you are in.
- You will be randomly selected to be in one of these two groups, and there is a 50% (or 1 in 2) chance of being assigned to either group, like the flip of a coin. Neither you nor the study team can choose your group.
- An inactive form of stimulation, or “sham,” will be used to find out if the active stimulation works better or is safer than receiving inactive stimulation for MDD.

Participant Population and Study Enrolment

This study will include 20 participants aged above 18 years old with a diagnosis of MDD. Participants will be enrolled in the Interventional Psychiatry Program at St. Michael’s Hospital, Unity Health Toronto.

Study Duration

Overall, this study will run for about twelve months. Your involvement in the research study will last for about three months.

Description of Research Activities

Research Procedures

The care that you receive for your condition will not be changed if you decide to participate in this study. All research interventions and activities will be in addition to usual care.

The FES will be administered using the Twin Stim® Plus Digital TENS/EMS stimulator. This is an electrical stimulation device which will deliver small amounts of electrical current to your facial muscles through electrodes attached to a mask. Each FES session will take place over a videoconference on the Zoom platform. A video camera, likely the webcam, will be used to record your facial expression during the study because the activation of particular muscles may later need

to be confirmed and analyzed. The video recordings are mandatory, and your smile will be detectable through the mask's hollowed-out area around the eyes and the mouth.

Research Questionnaires and Interviews

In order for us to gain a better understanding of the severity of your depressive symptoms, you will be asked to complete a few questionnaires and two interviews throughout your involvement in the study. Some of these questionnaires will be collected digitally through REDCap; a web-based platform that has fillable questionnaires for you to answer, while a member of the study team will ask you a series of questions verbally in the interview. Not all questionnaires and interviews will be conducted at every visit, and a schedule with the study activities and approximate duration of each visit is provided in the next section.
The Study Coordinator will ask you to complete 8 questionnaires using REDCap and 2 semi-structured interviews. Those marked with an asterisk (*) are semi-structured interviews asked verbally by the study team:

- The Mini-International Neuropsychiatric Interview (MINI)
- The Antidepressant Treatment History Form—Short Form (ATHF-SF).
- The Montgomery-Asberg Depression Rating Scale (MADRS)*
- The Hamilton Depression Rating Scale (HAMD)*
- The Quick Inventory of Depressive Symptomatology (QIDS)
- The General Anxiety Disorder Scale (GAD)
- The World Health Organisation-5 Well-Being Index (WHO-5)
- The Pittsburgh Sleep Quality Index (PSQI)

These questionnaires and interviews involve questions about your depressive symptoms and episodes which may seem personal or make you feel upset. In addition to this list, you will be asked to go through brief self-report scales on your expectations from FES and your experience during each FES visit via REDCap.
You may take as long as needed to complete the interviews and questionnaires, and you may skip any questions or questionnaires you do not wish to answer. If you have any questions or concerns while answering these questions, please talk to a study team member.

Mask Development

The manufacturing of the mask will need to be personalized so that we can properly target your facial muscles for electrical stimulation. An accurate 3D scanning of your face is a preliminary step to obtain the configuration of your facial features and to construct a 3D-printed mold that the customized, flexible mask will be casted onto. The scanning will happen during the first onsite visit, which will take about 20-30 minutes. A pilot FES will be done to ensure about the correct location of electrodes placed in the mask based on your face configuration. At the next on-site visit, you will receive the cast-molded mask. You will be given specific instructions on how to put on the mask and how to perform a facial expression known as the Duchenne smile. You will be allowed to practice with guidance from the investigator until the movement can be produced reliably. FES will

be applied at low intensities to your arm in order to show you what kind of sensations to expect before the stimulation is applied to your face.

Randomization

After your first on-site visit, you will be randomized. Randomization means that you are put into a group by chance, like flipping a coin. You will be assigned to one of two groups: one group will receive active FES, and the other will receive sham. Participants will be randomly assigned to receive 20 days (one 60-min session a day, 45 min for stimulation and 15 min for preparations) of active or sham FES. Sham intervention (as a placebo intervention) is an intervention using the same technology without using active stimulation. This method is commonly used for research comparison purposes in order to prove the efficacy of new potential treatments. Neither the PI nor the staff member conducting your weekly assessments will know what group you are in.

Collecting Information from medical records/health information

If you agree to participate in this study, the study team will collect the following health information from your Unity Health Toronto medical record:

- Psychiatric or medical conditions
- Current and past medications
- The presence of surgical implants
- Illnesses or psychiatric procedures that may influence the ability to participate in the study.

Summary

There is a total of 1 screening visit over the phone, 3 on-site visits, 20 home-based intervention visits, and 3 home-based follow-up visits:

- 1 screening visit over the phone
- 1 on-site mask development visit
- 1 on-site visit for the purpose of testing electrode placement, receiving the personalized mask and the FES device, instructions for use, and a webcam, and training on how to use the equipment.
- 20 intervention visits- 1 session per day, Monday to Friday, for 4 weeks
- 4 weekly follow-up visits over 4 weeks after the study (3 follow-up visits will happen over Zoom and the last follow-up visit will take place on-site).

As there are several visits in this study, the intervention schedules and assessment bookings can be e-mailed to you for your convenience; alternatively, we can also have a printout of all scheduled visits ready after you are enrolled.

MEDICATION CHANGE DURING STUDY:

You will be asked not to change any of your psychiatric medications or other forms of treatments (e.g., psychotherapy) for 4 weeks before, during, and 4 weeks after the course of 20 treatments if at all possible. If changes to treatment regimens or the introduction of psychotherapy are determined to be required as per your standard of care, the required changes will be made, and you will be withdrawn from the study.

Study Visits and Procedures

Screening Visit:

After indicating interest to a member of the study staff, you will be contacted by the study coordinator of this study. During a pre-screening telephone call, researchers will provide a brief summary of the study and any of your initial questions will be answered. If you are interested in continuing in the study, your verbal consent will be obtained before any other study activity and you will be asked to confirm your email address. Then you will be asked to complete a screening questionnaire via REDCap. The link to REDCap survey will be sent to the email address you provided. If you appear eligible, you will receive another phone call from the research coordinator to schedule your screening visit. Then, your screening visit will be scheduled. The reminder of visit will be sent via email or call at least 24 hours before this Visit. The written consent will be obtained at your first on-site visit.

The first study visit will be a telephone screening visit. This visit will involve an interview with the study psychiatrist to confirm your diagnosis and rule out other psychiatric diagnoses that might interfere with study intervention and confirm that you can safely undergo FES. The questions at the screening visit will help the researchers decide whether you are eligible to continue in this study.

This visit could take up to 90 minutes.

Mask development Visit:

In this on-site visit, you will be invited to the photo shooting room for the purpose of designing a personalized/portable mask with stimulation electrodes. The reminder of visit will be sent via email or call at least 24 hours before this Visit. Before taking the photos, you will be asked to take off any objects that may cause issues with capturing clear photos of your face (such as headbands, jewelry, reflecting make-up, long facial hair covering the cheeks, etc.). You will be seated on a chair with a suitable lighting environment. A softbox lighting setup will be used to allow for perfect photo shooting, as conventionally used in portrait photography studios. An average of 50 pictures will be taken, covering mainly the face and head areas from left to right and down to up. You might be asked to put on a rubber hair cap during the photo shoot, which might lead to better results when converting the 2D photos into a 3D scan during the image processing stage.
Following this, single-use FES electrodes (MyndTec) made of skin-safe materials will be placed on your face, and the FES will be tested to stimulate your target muscles. In order to determine the

proper electrode placement locations, 8 electrodes will be placed on your face: 2 electrodes under the left eye, 2 electrodes under the right eye, 2 electrodes on the left cheek, and 2 electrodes on the right cheek. We will aim to induce the desired muscle contraction with as little current as possible and with minimal discomfort to you. Once the electrode locations are determined, another picture will be taken so that the electrode placement will the same on your personalized mask.
The processing of 2D pictures into 3D profile, the 3D printing of the mold, and the casting of the flexible mask will be done offsite, and the entire process will take approximately 10-15 hours.

This visit could take up to 40 minutes.

Mask delivery Visit:

The second in-person study visit will be done to deliver the mold-casted mask to you. The reminder of visit will be sent via email or call at least 24 hours before this Visit. At this visit, you will also receive the preprogrammed stimulator with instructions and a webcam for the online monitoring during the intervention visits. The study team will provide verbal and written instructions on how to use the mask and the stimulator as well as how to perform a facial expression known as the Duchenne smile. We will attach the electrodes to the mask surface facing the skin and then run the cables for connection to the stimulation system. The mask will also be equipped with elastic fasteners to hold the mask on your face during the stimulation. The mask customized for you will now be ready for mounting on your face and performing the targeted stimulation.

This visit could take up to 60-90 minutes.

Baseline Visit:

A baseline visit will happen via videoconference before the first FES intervention visit. The reminder of visit will be sent via email or call at least 24 hours before this Visit. This visit will take about 45 minutes. A psychiatric interview will be administered by the study coordinator and the self-report questionnaires will be recorded in REDCap. Using questionnaires, the research team will assess your depression, sleep, and quality of life at baseline.

Intervention Visits 1-20:

The home-based study intervention will take place over 4 weeks, every day from Monday to Friday (20 visits total) over a videoconference on the Zoom platform. The reminder of visits will be sent via email or call at least 24 hours before each intervention Visit. On each day, you will have one session of FES for approximately 60 minutes (45 mins for stimulation and 15 mins for preparations) and a brief set of questions that will take an extra 10 minutes, will be answered in REDCap. Before each FES session, any cosmetics should be removed from your face prior to FES being applied. You will put on your mask and will be instructed on how to start the stimulation. Due to the customized design for each person with all components integrated into the mask, the process of wearing the

mask and using it should be very straightforward. You will place the mask on your face. According to the instruction you received at the mask delivery Visit and with the assistance of one of the study staff monitors, you will set the intensity of the device at the beginning of the intervention visit. There will be an external box with four switches for the four channels. For each stimulation, you will turn on the switch and adjust the stimulation intensities with the stimulator as instructed by the study staff. Once adjusting the intensity for that channel is finished, you can turn off the switch and move to the next channel. Once all 4 channels are adjusted, you can switch all of the channels on to start the stimulation for the session. After setting the stimulation intensity, you will be asked to remain awake, and be seated in a chair.

During the study intervention, you should be seated comfortably, and you will receive automated messages over your speakers to "smile & hold" precisely based on the intended pattern. The webcam will be used to video-record your smile patterns during the study because the activation of particular muscles may later need to be confirmed and analyzed. A physiotherapist or a trained staff will monitor the visit. The session will consist of alternating periods of FES and rest for 15-seconds each. Whenever the FES is on, you will be required to voluntarily produce the Duchenne smile. You will receive verbal reminders to do this. While the stimulator is active, you may experience tingling or pain on the skin or upper regions of the face near the site of stimulation. You may also experience other possible side effects, described in greater detail below.

You will be randomly assigned to self-deliver "active stimulation" or "sham stimulation". This will be pre-programmed into your take-home device and will be delivered automatically by the device once you set the intensity and press the "ON" button. You will be asked to voluntarily produce the Duchenne smile no matter which group you are assigned to.

Study intervention visits will last about 60 minutes, and visits 5, 10, 15, and 20 will last about 105 minutes since they include additional questionnaires (will be recorded in REDCap similar to the baseline visit).

Follow-up visits:

The study team will follow your progress with additional 3 videoconference appointments at 1, 2, 3 weeks after you complete your FES visits, and one onsite appointment at 4 weeks after you complete your FES visits. The reminder of visits will be sent via email or call at least 24 hours before each follow-up Visit. These visits will involve questionnaires (will be recorded in REDCap) and an interview similar to the baseline visit and will take about 45 minutes. You will return the stimulator, unused electrodes, and the webcam at the end of the study in the 4th follow-up visit, but you can keep the mask.

Table of Study Visits and Research Activities:

		Study Period																										
	Enrollment	Mask development	Allocation	Baseline	Post Allocation: Randomized Treatment Phase																				Follow up Period (With no intervention)			
Timepoint	-3	-2	-1	0	1	2	3	4	5	6	7	8	9	10	11	12	13	14	15	16	17	18	19	20	21	22	23	24
	**Enrollment**:																											
Eligibility	X																											
Demo	X																											
MINI	X																											
ATHF	X																											
MSF	X																											
MADRS	X																											
FES safety	X																											
Allocation			X																									
Mask design		X																										
Mask delivery			X																									
	**Intervention**:																											
A-FES (45 Min)					X	X	X	X	X	X	X	X	X	X	X	X	X	X	X	X	X	X	X	X				
B-Sham (45 Min)					X	X	X	X	X	X	X	X	X	X	X	X	X	X	X	X	X	X	X	X				
	**Assessment**:																											
HAM-D-17	X			X					X					X					X					X	X	X	X	X
QIDS-SR-16				X	X	X	X	X	X	X	X	X	X	X	X	X	X	X	X	X	X	X	X	X	X	X	X	X
AE (self-report)					X	X	X	X	X	X	X	X	X	X	X	X	X	X	X	X	X	X	X	X	X	X	X	X
GAD-7				X					X					X					X					X	X	X	X	X
WHO-5				X					X					X					X					X	X	X	X	X
PSQI				X					X					X					X					X	X	X	X	X
CONMED				X					X					X					X					X	X	X	X	X
FES Expectancy				X					X					X					X					X	X	X	X	X
FES Experience									X					X					X					X				

DEMO= Demographic form
MINI= Mini-international neuropsychiatric interview
ATHF= Antidepressant Treatment History Form
MSF= Medical history, Smoking history, Family history
MADRS= Montgomery-Åsberg Depression Rating Scale
FES= Functional Electrical Stimulation

HAM-D-17= Hamilton Depression Rating Scale – 17-Item
QIDS-SR-16=16-Item Quick Inventory of Depressive Symptomatology
AE= Adverse Event
GAD-7= Generalized Anxiety Disorder-7 Scale
WHO-5= World Health Organization-5 Well-Being Index
PSQI= Pittsburgh Sleep Quality Index
CONMED= Concomitant Medications

Participant responsibilities

It is important to remember the following if you choose to participate in this study:

- Ask your study team about anything that worries you.
- Tell study staff about any changes in your health.
- Tell study staff if you are considering any changes to your medications or doses.
- Tell study staff if you have changed any of your medications or doses.
- Tell study staff if you become pregnant during the study.
- Tell study staff if your depression becomes worse.
- Tell study staff if you are having thoughts about hurting yourself or anyone else.
- Tell your study team if you change your mind about being in this study.
- Tell your study team if you are considering enrolling in another study.
- You are not allowed to discuss the intervention with other participants of the study. You may be withdrawn from the study for doing so.

Potential Risks of Participating in This Study

As a result of your participation in this study, you are at risk for the side effects listed in this section. You should discuss these with the investigator and your regular doctor if you choose.

Potential Risks of Study Intervention

General Risk of Study Visits:
You may feel fatigued or uncomfortable during the study sessions. You may take breaks if needed.

Risks related to the FES intervention:

More Common

- Sensations under the electrodes
  - 20-70% of participants undergoing FES felt sensations under the electrodes, such as tingling or itching.
  - 10-20% of participants feel discomforts such as mild pain or burning.

These sensations usually resolve shortly after the start of stimulation. We turn the stimulation on slowly to keep these sensations to a minimum.

- Skin Irritation
  - The electrodes used may cause skin irritation or redness (30%)

- Other common side effects include:
  - Moderate muscle fatigue or muscle soreness (20 to 49%). Muscle soreness: not serious, reversible in 1-2 days
  - Headache (10-15%)
  - Difficulties in concentration (11%)

Less Common

- Nausea or nervousness (<5%)
  - If you feel dizzy, lightheaded, or that you might vomit, that study visit will be stopped for further examination by the study physician. The physician will decide whether you can continue the study.

Rare

- Seizure or any type of brain damage
  - A seizure is a convulsion where a person's body shakes. There is a theoretical risk of seizure or any type of brain damage from FES. It has never been observed in research studies involving FES, but it is possible.
  - If you have a seizure, you should call 911 immediately, and the study staff will give you instructions till they arrive.
- Muscle twitching or tingling sensation on your body.
- Ringing in the ear (<1%)
- Transient visual disturbance (2%)
  - Some participants have reported a sensation of feeling as if their vision is shaking or moving during the stimulation. This has gone away when the stimulation is stopped.

Tingling in upper extremities (<1%)

Potential Risks of Research Activities

Risks Related to Study Interviews/Questionnaires:

Self-report assessments contain questions regarding sensitive personal information. You may experience a negative reaction or feelings of distress when responding to some of the questions during the interview/in the questionnaires. If this happens, you can pause or stop your participation in the interview/questionnaire. You can also choose to withdraw from the study. The study team is available to discuss your concerns and/or to refer you to appropriate resources. This risk is necessary in order to assess your mood symptoms and associated psychopathology. You will be assured upon intake that only study personnel will see any rating form responses.

Risks related to the technologies for designing the mask:

The materials used for printing the FES mask are chosen to be compatible with human skin. Therefore, it is expected that there will be no skin sensitivity to the mask's material itself. However, we will monitor your face after experiencing multiple courses of stimulation. If any skin reaction (e.g., redness and bruising) is observed on your face, we will switch to other available compatible materials for the printing of the mask.

Reproductive Risks

For female participants who are able to conceive, there are no known risks to eggs (ova) or on an unborn baby (fetus) related to FES stimulation. However, there is always a possibility that if participants are pregnant, FES stimulation may have risks that we do not know about. For this reason, such participants should not participate in the study if they may be pregnant.

Appropriate family planning methods will be discussed with you and decided upon in consultation with the study doctor. If you are a female of childbearing potential and are not pregnant before starting the study, the study staff will ask you to confirm you are not pregnant and you are not planning to become pregnant throughout the duration of the study. If you become pregnant during your involvement in the study, you should immediately notify the investigators.

Potential Benefits

You may not benefit directly from participation in this study.
Your participation may help others in the future as a result of knowledge gained from the research.

Alternatives to Participation

You do not have to join this study to receive treatment for your condition. If you decline to be in this study, your care will not be affected. There are other options available to you that may help resolve your symptoms of depression and anxiety, such as psychotherapy or other medications.

In making your decision, you should keep in mind that being in a study is not a form of treatment and that participating in a study is not the same as being treated. Participation in this research study does not take the place of routine physical examinations or visits to your regular doctor. We

recommend that you discuss these and other options with the investigator and your regular doctor so that you can make a well-informed decision about participating in this study.

<u>Privacy and Confidentiality of Your Personally Identifiable Information and Study Data</u>

This section describes how your identifiable information and study data will be accessed, disclosed, and stored during this study. All people involved in the study are committed to respecting your privacy. Other than the individuals or groups described in this section, no persons will have access to your identifiable information without your consent, unless required by law.

Personally identifying information is any information that could be used to identify you and includes your name, address, date of birth, or health card number.

Study data is information that is generated by and/or collected for a study that has been stripped of personally-identifying information.

Protecting Your Privacy

The study personnel will make every effort to keep your personally identifying information private and confidential in accordance with all applicable privacy legislation, including the Personal Health Information Protection Act (PHIPA) of Ontario.

In addition to the study team, other authorized employees of Unity Health Toronto may have access to your personally identifying information so that they can carry out regulatory or institutionally required duties. Unity Health Toronto may also store personally identifying information that is collected or used for these duties for a period of time in accordance with regulations and institutional policies.

Any data collected for study purposes will be de-identified by using a unique study identification number instead of any of your personal identifiers. The study doctor at St. Michael's Hospital is in control of the key that links your study number to your personal information and will keep it stored separately from the study data. No personally-identifying information will be allowed off-site in any form unless required by law or as described in this consent form.

Medical Records

By signing this form, you are authorizing access to your medical records by the study personnel. The study team will also collect information from your medical record. The information that will be

collected is described in the Research Activities section. The study personnel will use this information to conduct the study.

You are also authorizing access to your medical records by representatives of the Unity Health Toronto Research Ethics Board and by applicable government regulatory authorities (e.g. Health Canada). Such access will only be used to verify the authenticity and accuracy of the information collected for the study, without violating your confidentiality, to the extent permitted by applicable laws and regulations.

Use of Email for Research

There are common risks of using email messaging to communicate:

- Information travels electronically and is not secure in the way a phone call or regular mail would be.
- If someone sees these emails, they may know that you are a participant in this study or see the health information included in the email.
- Emails may be read or saved by your internet or phone provider (i.e., Rogers, your workplace, “free internet” providers).
- Copies of an email may continue to exist, even after efforts to delete the email have been made.
- There is always a chance with any unencrypted email, however remote, that it could be intercepted or manipulated.

Do not use email and/or text messaging for medical emergencies. If you require immediate help, call your clinic or care provider, or seek emergency services.

Transfer of Personally Identifying Information Outside of Unity Health Toronto

The photos of your face taken during the mask development visit will be sent to a lab at the University of Toronto to develop the mask. This transfer will happen because a specific type of chemical component is needed to make the 3D mold. Also, a specific type of 3D printer is needed to develop the cast-molded mask.

The files will be physically brought over on a secured laptop where they are stored. At the University of Toronto, the pictures will be processed on a laptop with proper digital security. The laptop will be password-protected and is physically locked in a cabinet with a single key.

The pictures of your face will be deleted after the mold is 3D printed. The mold will be also destroyed immediately after printing the mask. The printed mask will be delivered to you. You can keep the mask after this study, but you will need to return any unused electrodes.

Personally Identifying Information and Study Data Storage and Retention

All personally identifying data used in this study will be securely stored. All the study data, except the images that will be captured to develop the mask and the videos that will be recorded during the stimulation visits, will be collected on paper forms and later transferred to an electronic database for analysis.

After printing the mask, the captured photos of your face will be destroyed. The captured photos will not be included in published documents or shown for educational purposes. The videos recorded during the intervention visits will be destroyed after the study team has analyzed the smile pattern. Video recordings will only to be viewed by the study personnel for quality assurance purposes. They will not be included in published documents or shown for educational purposes. Your research records will be kept for 7 years after study completion at St. Michael's Hospital in a highly secure and confidential manner.

As a reminder, study data is information that is generated by or collected for a study that has been stripped of personally-identifying information.

Study data will be securely stored at Unity Health Toronto. Study data will not be transferred outside of Unity Health Toronto and shared with others for purposes related to the conduct of this study.

Individual-level de-identified study data or results may also be used by this research team to answer similar questions and/or made available to scientific journals and their reviewers, other researchers inside or outside of Unity Health Toronto, public or private entities, or the public. Care will be taken to minimize the possibility of reidentifying you.

Personally identifying information collected for research purposes will be kept by the Principal Investigator and Unity Health Toronto for as long as required by Unity Health Toronto policy (currently 7 years after this study ends), at which point any documents with personally identifying information will be destroyed.

Online surveys and third-party website

The online survey is hosted by Research Electronic Data Capture (REDCap; https://www.project-redcap.org) software supported by Applied Health Research Centre (AHRC) at Unity Health Toronto (UHT). REDCap will be used for data collection and stores survey data on servers located in Canada. You will need to provide an email address to create an account. This system will be used to send scheduled questionnaires to you and store data. Data collected via surveys completed through

REDCap will be stored on the REDCap servers located at Unity Health Toronto.

Study Results and Study Registration

Results

The results of this study may be presented at a scientific conference or published in a scientific journal. If you are interested in obtaining the results of the study, you can contact the investigators or research team. We estimate that the results of the study will be available in 7 years.

Some scientific journals may require us to make the study data available to the journal, its reviewers, or to other researchers. You will never be personally identified in any publication, report, or presentation that may come from this study.

Registration

A description of this clinical trial will be available on http://www.ClinicalTrials.gov, as required by clinical trial regulations. This Web site will not include information that can identify you. At most, the Web site will include a summary of the results. You can search this Web site at any time.

The registration number for this study is ****.

Participation and Withdrawal

Voluntary Participation

Your participation in this study is voluntary. You may decide not to be in this study or to be in the study now and then change your mind later. You may leave the study at any time. If you choose not to participate, there will be no impact on the medical care received at, employment at, or other relationship with Unity Health Toronto now or in the future for you or your family.

We will give you new information that is learned during the study that might affect your decision to stay in the study.

Withdrawal from the Study

You may withdraw from the study at any time without giving a reason. You may withdraw from this study at any time without any effect on the medical care, employment or other relationship you or your family have at or with Unity Health Toronto.

Your participation in the study may be stopped without your consent for the following reasons:

- The research team decides to stop early

- Your physician feels that FES is causing you harm
- The research ethics board withdraws permission for this study to continue
- If it is discovered that you do not meet the eligibility requirements

Also, you might be discontinued from further participation if:

- You fail to adhere to the study procedure.
- You miss more than two intervention visits.
- You experience worsening depression, based on the study questionnaires.
- You meet any exclusion criteria (either newly developed or not previously recognized).

This study may be terminated by the study investigators or by the study sponsor at any time for any reason.

If you are withdrawn from this study or if this study ends early, a study team member will discuss possible next steps with you.

Continued Collection and Use of Your Data after Withdrawal

If you withdraw from the study, any data collected about you up to that time will still be used for analysis, and you will be asked to provide details about your experience with the FES one last time. No further data about you will be collected unless it is necessary to follow up on an adverse event that is not resolved at the time of your withdrawal. In the case of reporting unresolved adverse events, at the time of your withdrawal, you will be asked to participate in laboratory tests or medical examinations that the doctor considers necessary.

We may be required to retain the personally identifying information and study data that we have already collected until after the end of this study (described in the Privacy and Confidentiality section).

You will not have access to the study intervention after your involvement in the study.

Costs and Reimbursement

There is no cost to you for participation in this study. You will be reimbursed $30 per in-person visit, covering all travel expenses by TTC. You will receive a payment at each on-site visit, and no receipts will need to be provided.

If you withdraw from this study before completing it, you will receive compensation for the parts of the study that you have completed.

Compensation for Injury

If you are injured because of your participation in this study, medical care will be provided to you in the same manner as you would ordinarily obtain any other medical treatment. In no way does signing this form waive your legal rights nor release the study doctor(s), sponsor, or involved institution(s) from their legal and professional responsibilities.

Rights as a Participant

If you are harmed as a direct result of taking part in this study, all necessary medical treatment will be made available to you at no cost. By signing this form, you do not give up any of your legal rights against the investigators, sponsor, or involved institutions for compensation, nor does this form relieve the investigators, sponsor, or involved institutions of their legal and professional responsibilities.

New Information About the Research Study

During the study, we may make changes to the study. We may also learn new things about the study that you may need to know. Some of the new information or changes might affect your decision to take part in the study. If so, you will be notified about the new or changed information in a timely manner and we will ask you if you consent to remain in the study. You may be asked to sign a new consent form at that time.

New Information About Your Health (Incidental Findings)

The tests or procedures that we do during this study might reveal medical information about you that is not part of the objectives of this study but may be relevant to your health. This type of medical information is called an incidental finding. Some incidental findings could be related to treatable conditions, or they could be related to factors that may affect your current or future health care. With your consent, we will communicate all medically actionable incidental findings to you.

Research Ethics Board Contact

If you have any questions regarding your rights as a research participant, you may contact the Unity Health Toronto Research Ethics Board Office at 416-864-6060 ext. 42557 during business hours (9:00 am to 5:00 pm). Unity Health Toronto is a health network that includes Providence Healthcare, St. Joseph's Health Centre, and St. Michael's Hospital.

The Unity Health Toronto Research Ethics Board is made up of a group of scientists, medical staff, and individuals from other backgrounds (including law and ethics) as well as members from the community. The Board is established by Unity Health Toronto to review studies for their scientific and ethical merit. The Board pays special attention to the potential risks and benefits to the research participant, as well as the potential benefit to society.

Study Contacts

If at any time during this study you have questions about the study or the research activities, you should contact the Principal Investigator, **Dr. Venkat Bhat** at (416) 864-6060 x76404.

If you have any questions or concerns that require urgent attention outside of the study hours of availability, or would like to speak to the study team for any reason, you can contact: **Dr. Venkat Bhat** at (416) 864-6060 x76404 or **Mrs. Fatemeh Gholamali Nezhad** at (416) 360-4000 x 40675.

In case of emergency, please go to the nearest emergency department or call 911 for assistance. Let them know that you are in a study, and the Principal Investigator's name.

You will be given a signed copy of this consent form.

Signature Page: Documentation of Informed Consent

Study Title: "Take-Home" Functional Electrical Stimulation for Depression: Prototype Development and Proof of Concept Clinical Trial

Participant Statement of Consent

By signing this consent form, I acknowledge that:

- This research study has been sufficiently explained to me both verbally (prior to this visit) and in person, and my questions have been answered to my satisfaction.
- I have been informed of the alternatives to participation in this study.
- I know that I have the right not to participate and the right to withdraw from this study without affecting the quality of medical care at Unity Health Toronto for me and for other members of my family.
- The potential risks and benefits (if any) of participating in this research study have been explained to me.
- I have been told that I have not waived my legal rights nor released the investigator, sponsor, or involved institutions from their legal and professional responsibilities.
- I know that I may ask, now or in the future, any questions I have about this study.
- I have been told that records relating to me and my care will be kept confidential and that no personal information will be disclosed without my permission unless required by law.
- I have been given sufficient time to read the information in this consent form.
- I will be given a signed and dated copy of this consent form.

I consent to participate in this study.

______________________	______________________	__________
Participant name (print)	Participant/Substitute decision-maker signature	Date

Please initial one of the boxes below to indicate whether or not you want to be informed of all medically actionable incidental research findings.

	**YES**, I agree to be told about any medically actionable incidental findings.
	**NO**, I do not want to be told about medically actionable incidental findings.

I have explained to the above-named participant the nature and purpose, the potential benefits, and possible risks of participation in this research study. All questions that have been raised about this study have been answered.

______________________	______________________	__________
Name of person obtaining consent (print)	Signature of person obtaining consent	Date

Complete the following section only if the participant has verbally consented:

- The consent form was explained to, and apparently understood by, the participant, _________________, and
- Informed consent was freely given by the participant

☐ The consent form was read to the participant and consent was received verbally via _______________________ (telephone, Zoom Healthcare, etc.). The person signing below attests that the study as set out in this form was accurately explained to the participant, and any questions have been answered. The participant will be mailed or emailed a copy of the consent form for their records.

______________________________	______________________________	______________
Name of person obtaining consent, Role (print)	Signature of Person Conducting the Consent Discussion	Date
